# Preparation of PAN@TiO_2_ Nanofibers for Fruit Packaging Materials with Efficient Photocatalytic Degradation of Ethylene

**DOI:** 10.3390/ma12060896

**Published:** 2019-03-18

**Authors:** Zhu Zhu, Ye Zhang, Yibo Zhang, Yanli Shang, Xueji Zhang, Yongqiang Wen

**Affiliations:** 1 Beijing Key Laboratory of Bioengineering and Sensing Technology, School of Chemistry and Bioengineering, University of Science & Technology Beijing, Beijing 100083, China; zhuzhu@ustb.edu.cn (Z.Z.); zhangxueji@ustb.edu.cn (X.Z.); 2 College of Chemistry & Environmental Science, Hebei University, Baoding 071002, China; 18330117319@163.com (Y.Z.); ybzhang0123@163.com (Y.Z.)

**Keywords:** electrospun nanofiber, TiO_2_, photocatalytic activity, ethylene degradation, tomato ripening

## Abstract

Ethylene causes faster deterioration of perishable crops during postharvest transportation and storage. The present study aimed to develop TiO_2_-coated nanofibers with efficient photocatalytic activities to enhance the degradation of fruit-emitted ethylene. The consecutive electrospinning of polyacrylonitrile (PAN) and TiO_2_ deposition was successfully performed to produce PAN@TiO_2_ nanofibers. The scanning electron microscopy results indicate the uniform distribution of TiO_2_ nanoparticles on the surface of the PAN nanofiber. The PAN@TiO_2_ composite nanofibers exhibited enhanced photocatalytic activity for ethylene degradation under low-intensity UV light irradiation. Furthermore, a tomato fruit-ripening test confirmed the effectiveness of the PAN@TiO_2_ nanofibers. The PAN@TiO_2_ nanofibers exhibited effective ethylene degradation and slowed the color shift and softening of the tomatoes during storage. The results suggest great potential for use of the PAN@TiO_2_ composite nanofibers as ethylene scavenging packaging material for fresh fruits and vegetables.

## 1. Introduction

Ethylene is a natural aging hormone that enhances the decay of perishable crops during postharvest transportation and storage. Hence, removing ethylene from the environment surrounding fresh produce plays a pivotal role in prolonging their shelf life and reducing postharvest losses [1]. Currently, active packaging, which involves the addition of active component in packaging systems, is widely implemented to maintain or extend food product qualities and shelf lives [2]. Several ethylene scavenging packaging systems have been developed for fruits and vegetables. Potassium permanganate (KMnO_4_) or activated carbon is used in the form of sachets [3,4]; and clays and halloysite nanotubes are incorporated into films as ethylene scavenging materials [5,6]. However, these oxidizers and absorbers are limited by waste disposal and limited adsorption capacity [7]. Emerging technologies based on photocatalytic oxidation of ethylene offer an alternative approach that could overcome these disadvantages. 

The photocatalytic oxidation of ethylene involves exposure to ultra violet (UV) radiation and the use of a catalyst such as titanium dioxide (TiO_2_). In general, reactive oxygen species (ROS) are produced on the TiO_2_ surface following exposure to UV light, thereby further oxidizing ethylene to carbon dioxide and water [8]. In the past decade, several composite films containing TiO_2_ to remove ethylene at different UV light intensity ranging from 42 μW cm^−2^ to 2.5 mW cm^−2^ have been reported. Tanaka et al. [9] prepared an adsorbents-embedded TiO_2_ film by sol-gel method. Maneerat and Hayata [10] used a polypropylene film coated with TiO_2_ as an ethylene scavenger packaging material. A chitosan–TiO_2_ nanocomposite film was also fabricated to prolong shelf life of tomato fruit [11]. 

Nanofibers offer some advantages over ordinary films including high surface-to-volume ratio, nanoporous structures and easy encapsulation of active agents [12]. Electrospinning provides a quick approach to fabricating continuous nanofibers and has been used to prepare inorganic nanoparticles in polymers. In our previous study, TiO_2_-embedded nanofibers were prepared by adding TiO_2_ nanoparticles into the polymer solution via one step electrospinning [13]. However, the homogeneous dispersion of nanoparticles in a polymer matrix is challenging due to the resulting agglomeration of nanoparticles and the high viscosity of polymers. Furthermore, the presence of a polymer layer surrounding the nanoparticles limits the interaction between the particle surface and oxygen or water. 

To address these problems, researchers have deposited inorganic nanoparticles on the surface of the nanofiber. For example, Hong et al. [14] fabricated PVA/ZnO composites via electrospinning followed by an in situ hydrolysis process; the ZnO nanocrystals were formed on the fiber surface with high density. Gupta et al. [15] achieved TiO_2_ deposition onto polylactic acid (PLA) nanofibers via hydrothermal synthesis and electrospinning, of which the resulting PLA/TiO_2_ nanofibers exhibited excellent UV absorption properties. 

The present study consecutively performed polyacrylonitrile (PAN) electrospinning followed by TiO_2_ deposition to produce PAN@TiO_2_ composite nanofibers, of which the TiO_2_ nanoparticles were assembled on the surface of the fiber rather than within the nanofibers to enhance its photocatalytic activity for the degradation of fruit-emitted ethylene. The morphology and the crystallinity of the produced composite nanofibers were investigated. The photocatalytic activity of the composite nanofibers in the degradation of ethylene was determined. The results show that the surface of the composite was coated with a larger amount of TiO_2_ nanoparticles, which could effectively increase the contact area between nanoparticles and ethylene and improve the degradation efficiency of ethylene. The composite nanofibers were then used to retard tomato fruit ripening during postharvest storage. 

## 2. Materials and Methods

### 2.1. Materials

Ammonium sulfate ((NH_4_)_2_SO_4_), titanium tetrachloride (TiCl_4_), dimethylformamide (DMF), and ammonia (NH_3_·H_2_O) were purchased from Beijing Chemicals (Beijing, China). PAN (polyacrylonitrile, M_w_ = 150,000) was obtained from Aladdin Reagent (Shanghai, China). Ethylene (99.995%) was obtained from Beijing ZG Gas Science & Technology Co., Ltd (Beijing, China). The tomatoes were harvested at the mature green stage from a greenhouse and sorted according to color and size without physical injuries or decay. All of the chemicals were of analytical reagent grade. 

### 2.2. Fabrication of PAN Nanofibers

PAN was dissolved in DMF to produce a polymer solution that was then loaded into a syringe equipped with a stainless-steel needle. The samples were collected using an aluminum sheet at a distance of 17 cm from tip to collector. The polymer solution was pumped at a constant rate of 0.5 mL/h, and at a voltage of 24 kV. The nanofibers were then dried overnight in a vacuum oven to allow solvent evaporation. 

### 2.3. TiO_2_ Deposition on Nanofibers

The PAN@TiO_2_ nanofibers were prepared following the methodology described by Shi et al. [16]. In an ice-water bath, 2.7 mL of TiCl_4_ was slowly added into a flask containing 45 mL of a (NH_4_)_2_SO_4_ solution (1 M) under vigorous stirring. The solution was then slowly heated to 90 °C, and a 0.2 g PAN nanofiber was immersed in this solution. The mixture was reacted at 90 °C for 1 h, and the pH of the solution was then adjusted to 7 via the addition of 2 M NH_3_·H_2_O. The solution mixture was cooled to room temperature, after which the nanofiber was removed, and washed three times with deionized water and vacuum-dried overnight at 30 °C.

### 2.4. Characterization of the Nanofiber

The PAN and PAN@TiO_2_ microstructures were characterized via scanning electron microscopy (SEM, SUPRA55, ZEISS, Berlin, Germany) and transmission electron microscopy (TEM, HT7700, Hitachi, Tokyo, Japan). The elemental composition of the nanofibers was confirmed by energy dispersive X-ray spectroscopy (EDS, XFlash 5010, Bruker, Berlin, Germany). The crystalline structure of the PAN@TiO_2_ nanofibers was examined with X-ray diffraction (XRD, D8 ADVANCE, Bruker, Berlin, Germany) using Cu Kα radiation (λ = 1.540 Å). The infrared spectra of the PAN and PAN@TiO_2_ nanofibers were recorded using a Fourier transform infrared spectrometer (FTIR, Nicolet Nexus 670) (Thermo Nicolet Corporation, Nikolai, America) in the range from 500 cm^−1^ to 4000 cm^−1^. UV-vis diffuse reflectance spectra of nanofiber were recorded on a UV-Vis spectrophotometer (TU-1901, Purkinje General, Beijing, China). The nanofiber samples were mixed with BaSO_4_ and compressed into a tablet. BaSO_4_ was used as a reflectance standard.

### 2.5. Photocatalytic Degradation of Ethylene

Ethylene degradation was carried out in a 5.4-L reactor under a UV lamp (16 W) (Haimen Kylin-Bell Lab Instruments Co., Ltd., Nantong, China), which had an intensity of 2.9 μW cm^−2^. The nanofibers were attached to the inner surface, and rubber plugs were used to plug the inlet and outlet.

One hundred parts per million of ethylene was then injected into the reactor, and the gaseous mixture was homogenized with a fan. After achieving steady-state conditions, the initial concentration of ethylene was measured with a handheld ethylene analyzer (F-950, CID Bio Science Inc. Shanghai, China) that automatically collected headspace samples. Then, the UV light was turned on, and the ethylene concentration was measured every 5 h. The ethylene degradation C (%) was calculated as follows:

$$C=\frac{\left(C_{i}-C_{f}\right)}{C_{i}}\times 100\%$$

where C_i_ and C_f_ are the initial and final concentrations of ethylene, respectively. The results were expressed as a percentage of ethylene degradation.

### 2.6. Fruit Ripening Test

Tomatoes were randomly distributed into two groups and placed in plastic boxes that are open at the top, specifically three boxes per group and eight fruits per box. Tomatoes were identical in each box. The boxes of the first group were fully covered with a layer of polypropylene (PP) film (25 μm thick, 72% transmittance at a wavelength of 365 nm) and served as the control. The boxes of the second group were fully covered with a layer of PAN@TiO_2_ nanofiber and a layer of PP film. The UV lamp was suspended above the boxes, the intensity was measured using a radiation meter, and was adjusted to 2.9 μW cm^−2^ by adjusting the distance between the light and the boxes. All of the boxes were stored at room temperature (22 °C) under UV irradiation until the fruits in the control groups were fully ripe. To determine the ethylene production, four fruit from each box were sealed in a gas-tight container for 3 h, and the concentration was measured as described above. The fruit firmness was measured with a digital fruit firmness tester (GY-4, Zhejiang Top Instrument Co., Ltd. Hangzhou, China) that was fitted with a cylindrical plunger (diameter, 3.5 mm). Each assay had three replicates, and the experiment was performed in triplicate.

## 3. Results and Discussion 

### 3.1. Characteristics of the PAN@TiO_2_ Nanofibers

**Figure 1:**
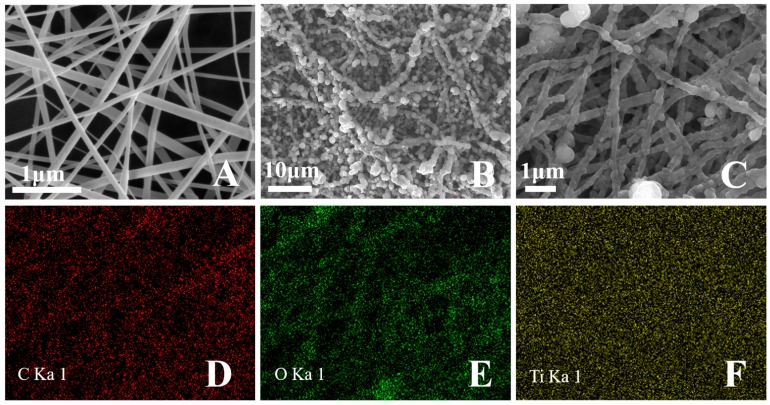
Scanning electron microscopy (SEM) images of: (**A**) electrospun PAN nanofibers; (**B**) PAN@TiO_2_ nanofibers at low magnification; and (**C**) PAN@TiO_2_ nanofibers at high magnification. EDS mapping images of PAN@TiO_2_ nanofibers: (**D**) carbon; (**E**) oxygen; and (**F**) titanium.

PAN is the widely used polymer matrix due to its high mechanical properties and chemical inertness [17]. The PAN nanofibers had smooth surfaces and nanometer-scale diameters (Figure 1A). TiCl_4_ served as the Ti precursor and the addition of ${\mathrm{SO}}_{4}^{2-}$ promoted the formation of anatase TiO_2_. TiCl_4_ followed a three-step hydrolysis mechanism, and the TiO_2_ precipitate was collected after temperature increased to 95 °C [18]. The TiO_2_ precipitate was positively charged due to the lower pH level of the mixed solution (pH < 1) [19]. Therefore, TiO_2_ with positive charges was attracted by the nitrile group of PAN with electronegativity to form nucleation sites. Enhanced TiO_2_ growth was observed on the nucleation sites following the addition of dilute NH_3_·H_2_O. The morphology of the PAN@TiO_2_ nanofibers is detailed in Figure 1B,C. The TiO_2_ nanoparticles were immobilized on the surface of the PAN nanofiber. The images demonstrate that the PAN nanofibers were successfully decorated with TiO_2_. The elemental composition of PAN@TiO_2_ nanofibers was confirmed by EDS mapping analysis. The images show that the sample contained C, O, and Ti elements, and the Ti element was well dispersed throughout the nanofiber (Figure 1D–F).

**Figure 2:**
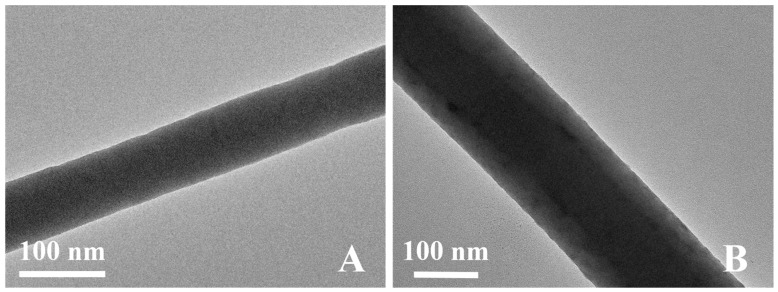
Transmission electron microscopy (TEM) images of: (**A**) electrospun PAN nanofibers; and (**B**) PAN@TiO_2_ nanofibers.

The microstructures of the nanoparticle-modified nanofiber were further characterized by TEM. Figure 2B presents the uniform distribution of the TiO_2_ nanoparticles along the nanofibers. This confirmed the continuous coating of TiO_2_ on the PAN nanofiber. 

**Figure 3:**
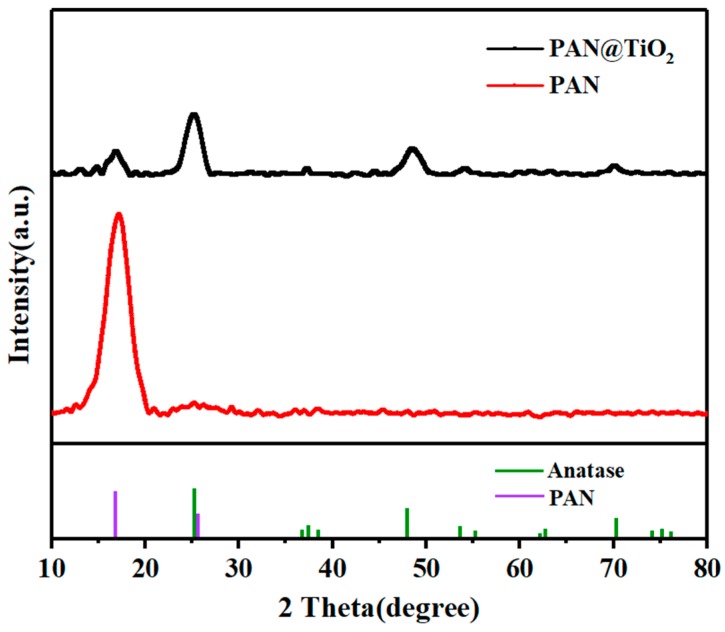
X-ray diffraction (XRD) patterns of PAN and PAN@TiO_2_ nanofibers.

The crystalline structure of the synthesized TiO_2_ was examined by XRD (Figure 3), in which a peak at 17° was observed, indicating the presence of PAN [20]. Other diffraction peaks were well indexed to anatase TiO_2_, suggesting that the synthesized TiO_2_ nanoparticles on the nanofibers were in the anatase phase. The crystal phase may have affected the photocatalytic efficiency, given that the anatase phase manifests a higher photocatalytic activity compared to the rutile and brookite phases [21]. For crystalline structure control, ${\mathrm{SO}}_{4}^{2-}$ was bridged by Ti^4+^ to help form anatase phase TiO_2_; otherwise, TiO_2_ had both rutile and anatase phase [18]. Moreover, the average crystallite size of TiO_2_ was calculated by the Scherrer equation: D = 0.89 λ/(*β* cos *θ*), where λ is the X-ray wavelength, λ = 0.154056 nm, θ is the Bragg’s diffraction angle, and β is the full width at half maximum. When β is 0.015 rad and θ is 12.67°, the crystallite size of TiO_2_ is 9.1 nm. It has been demonstrated that the nanoparticles in the range of 1–10 nm exhibit excellent catalytic characters owing to the quantum size effect [22,23].

**Figure 4:**
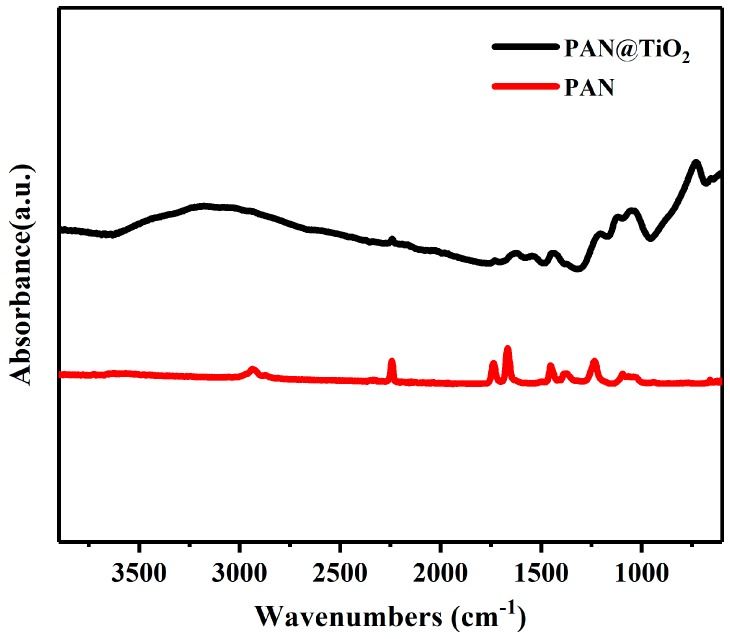
Fourier transform infrared spectrometer (FTIR) spectra of PAN and PAN@TiO_2_ nanofibers in the range of 4000 cm^−1^ to 500 cm^−1^.

The FTIR spectra of the PAN and PAN@TiO_2_ nanofibers were analyzed to examine the conversion of precursor chemical structure of the polymers (Figure 4). The FTIR spectra of the PAN nanofibers presented characteristic peaks at 2928, 2240, 1738, 1454, 1240 and 1033 cm^−1^, corresponding to CH stretching, C≡N stretching, C=O stretching, CH bending, CH rocking vibrations, and C=O rocking vibrations. After coating with TiO_2_, the spectrum of nanofibers showed distinct changes. Observed peaks ranging from 750 to 900 cm^−1^ corresponded to Ti-O stretching vibration. Similarly, an absorption peak observed at 3200 cm^−1^ suggested OH stretching vibration. Some water molecules in the air may have been adsorbed onto the surface of TiO_2_, such that the absorbance peak of hydroxyl can be attributed to the presence of Ti–OH on the surface of TiO_2_ [24]. These results demonstrate that TiO_2_ were successfully deposited onto PAN nanofibers, and the structure of TiO_2_ was not changed.

**Figure 5:**
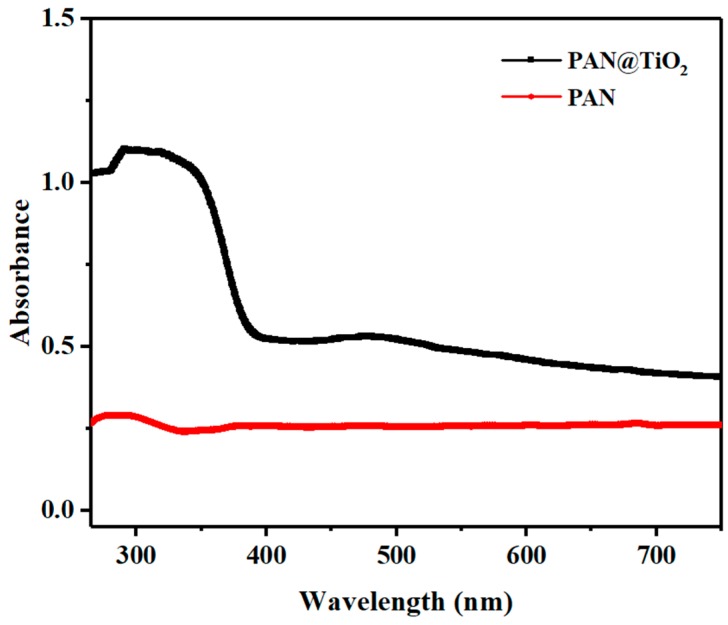
UV–vis diffuse reflectance spectra of PAN and PAN@TiO_2_ nanofibers.

UV absorption spectra of PAN and TiO_2_-coated nanofibers were recorded to evaluate their photoactive nature (Figure 5). Pure PAN nanofibers exhibited no absorption in UV region, while the TiO_2_-coated nanofibers had great absorption in the range of UV spectrum. The result are consistent with a previous study showing that TiO_2_ nanotubes absorbed UV light in the 300–400 nm range [25].

### 3.2. Photocatalytic Degradation of Ethylene

**Figure 6:**
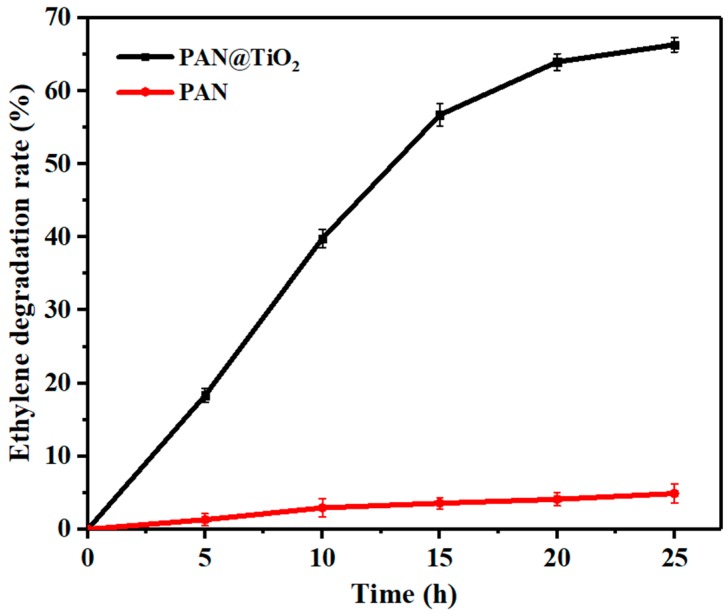
Photocatalytic degradation of ethylene using PAN@TiO_2_ nanofibers under UV irradiation. The data represent the means of three independent experiments. The error bars indicate the standard deviation of the mean.

The ethylene degradation efficiency of the PAN@TiO_2_ nanofibers were measured under UV illumination to evaluate their photocatalytic behavior. Under UV light, electrons on the TiO_2_ surface are promoted and transferred from valence band to the conduction band, leaving behind holes in the valence band. These electron–hole pairs are highly charged and can initiate reduction and oxidation reaction. In the presence of air and water, hydroxyl radicals and superoxide ions are generated to oxidize ethylene into carbon dioxide and water [8]. As indicated in Figure 6, ethylene degradation of PAN@TiO_2_ nanofibers was positively correlated with the UV irradiation time. Approximately 65% of the ethylene was degraded within 25 h. In contrast, ethylene content decreased slightly for pure PAN nanofibers, which was probably because the nanofibers with nanoporous structures and large surface area adsorbed a small amount of ethylene. The results demonstrate that TiO_2_-coated PAN nanofibers showed much higher photocatalytic activity than the TiO_2_-embedded nanofibers [13]. The well-separated TiO_2_ on the surface of the PAN nanofibers could be easily accessible to oxygen, water, and ethylene surrounding the nanoparticles. Furthermore, the homogeneous distribution throughout the nanofiber could enhance the contact area with UV light to remove ethylene [26].

### 3.3. Tomato Ripening Test

**Figure 7:**
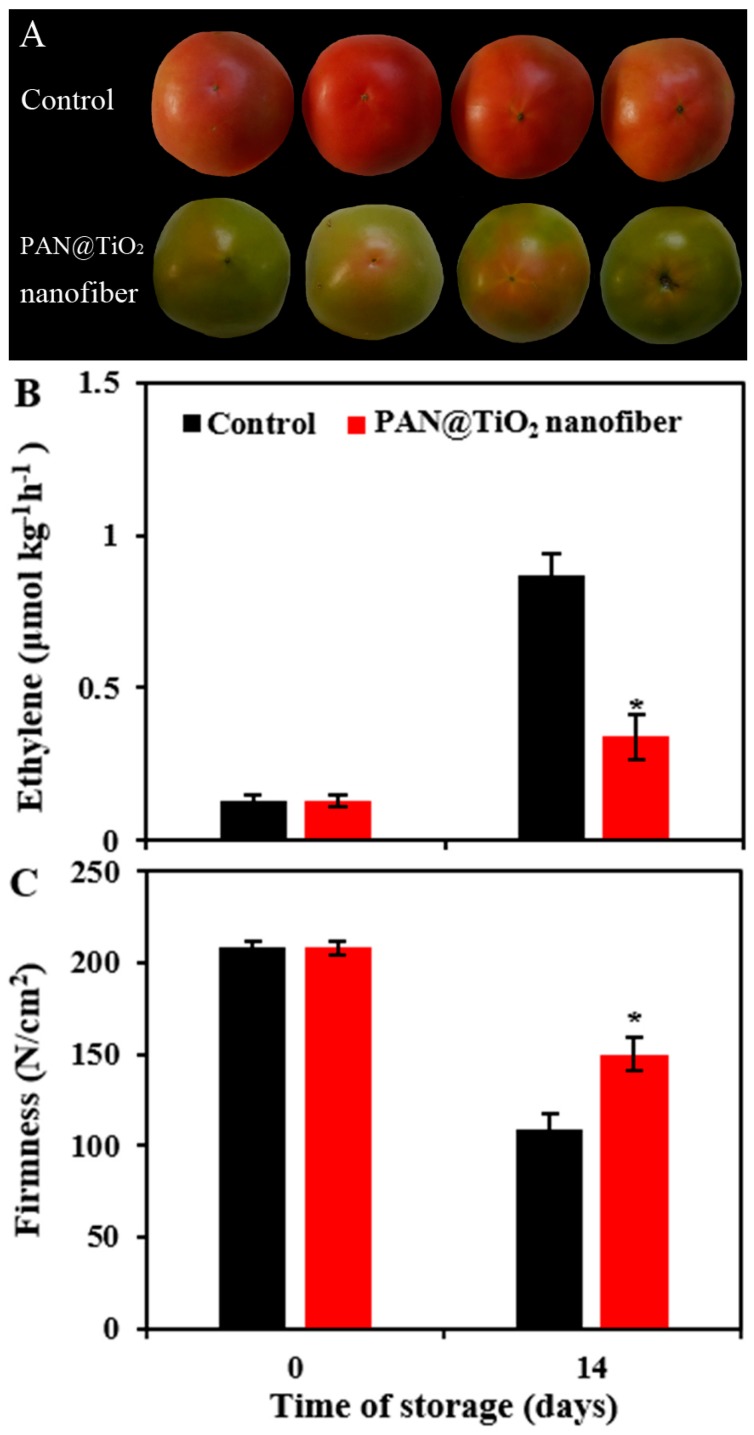
Photographs (**a**); ethylene production (**b**); and firmness (**c**) of tomatoes stored for 14 days covered with PP film (control) or PP film and PAN@TiO_2_ nanofibers. The data represent the means of three independent experiments. The error bars indicate the standard deviation of the mean. Asterisks indicate significant differences between the control and nanofiber-covered fruit according to the Student’s *t*-test (*P* < 0.05).

The photocatalytic activity of the PAN@TiO_2_ nanofibers were further evaluated using a tomato fruit-ripening test, wherein the significant increase in ethylene production was observed and color changes from green to red were monitored. Figure 7A shows images of the fruits coated with a layer of PAN@TiO_2_ nanofiber and a layer of PP film as well as fruits coated only with a layer of PP film following 14 days of storage at room temperature. Tomatoes coated with PP films turned red, while tomatoes coated with PAN@TiO_2_ nanofibers and PP films retained their initial green coloring. The control fruit exhibited rapid ethylene production after 14 days of storage. However, the fruits covered with PAN@TiO_2_ nanofibers showed remarkably lower ethylene levels (Figure 7B). The control fruits also exhibited a sharp decrease in firmness, while fruits covered with PAN@TiO_2_ nanofibers were much firmer (Figure 7C). Ethylene induces the expression of many ripening-related genes during tomato ripening, such as phytoene synthase (PSY) and polygalacturonase (PG). This leads to lycopene accumulation and cell wall metabolism [27]. Therefore, removal of ethylene from the surrounding atmosphere could delay color change and softening of fruit. These results demonstrate the effectiveness of the PAN@TiO_2_ nanofibers in retarding fruit ripening by the degradation of tomato-emitted ethylene.

The implementation of TiO_2_ films for the photocatalytic degradation of ethylene in postharvest fruits have been minimally reported. Maneerat and Hayata [10] reported that TiO_2_-coated PP film could decompose ethylene in packaged tomatoes under UV light at 1.5 mW cm^−2^. Kaewklin et al. [11] employed chitosan and a TiO_2_ nanocomposite film to delay tomato ripening by the application of a UV light at an intensity of 42 μW cm^−2^. In our study, a low intensity of 2.9 μW cm^−2^ was applied for the fruit-ripening test, and the PAN@TiO_2_ nanofiber exhibited beneficial effects on extending the shelf life of tomato fruit. This suggested that the nanofiber was highly efficient for photocatalytic degradation of ethylene and requires little energy. It has been reported that the photocatalytic process also removed acetaldehyde, ethanol, and off-flavors generated by red tomatoes during storage [28]. Although impact on other climacteric fruit is still unclear, and the largescale application still need further investigation, the PAN@TiO_2_ nanofiber could be used to remove ethylene from storage and prolong the shelf life of fresh produce.

## 4. Conclusions

The present study performed the consecutive electrospinning of PAN and TiO_2_ deposition to prepare PAN@TiO_2_ nanofibers with uniformly distributed TiO_2_ nanoparticles on the nanofiber surface. TiO_2_-coated PAN nanofibers showed much higher photocatalytic activity for ethylene degradation than the TiO_2_-embedded nanofibers under low-intensity UV light irradiation (2.9 μW cm^−2^). The PAN@TiO_2_ nanofibers also exhibited effectiveness in decomposing ethylene emitted by tomatoes and delayed fruit softening and color change during storage at room temperature. Therefore, the PAN@TiO_2_ nanofibers has great potential as an ethylene scavenging packaging material for fresh produce during postharvest transportation and storage.
